# Automatic Detection of Granuloma Necrosis in Pulmonary Tuberculosis Using a Two-Phase Algorithm: 2D-TB

**DOI:** 10.3390/microorganisms7120661

**Published:** 2019-12-07

**Authors:** Pelin Kus, Metin N. Gurcan, Gillian Beamer

**Affiliations:** 1 Department of Research, Development and Technology, Republic of Turkey Ministry of National Defence, 06100 Ankara, Turkey; pelinkus1@gmail.com; 2 Department of Internal Medicine, School of Medicine, Wake Forest University, Winston-Salem, NC 27109, USA; mgurcan@wakehealth.edu; 3 Department of Infectious Disease and Global Health, Cummings School of Veterinary Medicine, Tufts University, North Grafton, MA 01536, USA

**Keywords:** tuberculosis, granuloma, necrosis, algorithm, machine learning

## Abstract

Granuloma necrosis occurs in hosts susceptible to pathogenic mycobacteria and is a diagnostic visual feature of pulmonary tuberculosis (TB) in humans and in super-susceptible Diversity Outbred (DO) mice infected with *Mycobacterium tuberculosis.* Currently, no published automated algorithms can detect granuloma necrosis in pulmonary TB. However, such a method could reduce variability, and transform visual patterns into quantitative data for statistical and machine learning analyses. Here, we used histopathological images from super-susceptible DO mice to train, validate, and performance test an algorithm to detect regions of cell-poor necrosis. The algorithm, named 2D-TB, works on 2-dimensional histopathological images in 2 phases. In phase 1, granulomas are detected following background elimination. In phase 2, 2D-TB searches within granulomas for regions of cell-poor necrosis. We used 8 lung sections from 8 different super-susceptible DO mice for training and 10-fold cross validation. We used 13 new lung sections from 10 different super-susceptible DO mice for performance testing. 2D-TB reached 100.0% sensitivity and 91.8% positive prediction value. Compared to an expert pathologist, agreement was 95.5% and there was a statistically significant positive correlation for area detected by 2D-TB and the pathologist. These results show the development, validation, and accurate performance of 2D-TB to detect granuloma necrosis.

## 1. Introduction

Tuberculosis (TB) is diagnosed in 9–10 million human patients and causes 1–2 million deaths each year, surpassing mortality due to HIV/AIDS [1]. Most human patients develop pulmonary TB, with disease restricted to the lungs following airborne infection with *Mycobacterium tuberculosis*. Infection incites complex host responses involving epithelial cells, dendritic cells, monocytes, macrophages, myeloid-derived suppressor cells, neutrophils, antigen-independent and antigen-specific lymphocytes, plasma cells, stem cells, stromal cells, and many effector molecules [2,3,4,5,6,7,8]. Cells recruited to the lungs form multiple lesion types, including diffuse lipid pneumonia, bronchiolar obstruction, and granulomas in humans and in experimental animal models [9,10,11,12,13,14,15,16,17]. These different lesions have different functions and outcomes for bacilli and lung tissue [18,19] although detailed structure-function relationships at the cell, molecule, and gene levels remain to be determined. Here, we focus on automatic detection of intra-granuloma cell-poor necrosis because granuloma necrosis is a diagnostic visual feature of pulmonary TB in clinical and research settings. Further, granuloma necrosis is central to TB disease pathogenesis in susceptible hosts.

Much research has identified pathways that trigger necrosis, for example: Intracellular bacterial replication and macrophage lysis; hypersensitivity to *M. tuberculosis* cell wall components and induction of necrotizing lipid pneumonia; vascular thrombosis resulting in infarction and necrosis due to hypoxia [11,16,20,21,22,23,24,25,26,27]. These events converge on two visual patterns of necrosis in histological tissue sections, as interpreted by pathologists using light microscopes. One pattern contains abundant nuclear and cellular debris, recognized by nuclear pyknosis (shrinking), karyorrhexis (fragmentation), karyolysis (dissolution) and variably bright pink eosinophilic cytoplasm [28]. A second pattern of necrosis is recognized by loss of differential staining, i.e., affected tissue appears relatively homogenous and pink with minimal nuclear debris, and few viable stromal or inflammatory cells [28,29,30]. Because cell, granuloma, and lung tissue necrosis cause symptoms in pulmonary TB patients and contribute to transmission of *M. tuberculosis* bacilli [10,30], we developed and validated a method that allows investigators to automatically detect necrosis. We focused on the pattern of cell-poor necrosis within *M. tuberculosis* granulomas.

Previous approaches to distinguish necrotic from non-necrotic regions using computer-aided image analysis and machine learning algorithms can be grouped into four categories: (i) color statistics and local binary patterns [31], (ii) visual patterns [32,33,34], (iii) kernel methods [35], and (iv) neural networks [36]. Homeyer et al. [31] achieved best accuracy and computation time using color and texture features from Red Green Blue (RGB) and Hue Saturation Value (HSV) color channels. They used 4 sets of liver images and features from HSV color channels and a Random Forest classifier to obtain >88% accuracy in detecting hepatic necrosis. Cruz-Roa et al. [32] used a different approach, extracting visual patterns from normal and necrotic tissues using a bag of features-based codebook. This codebook comprises of luminance values of block pixels, series of difference of Gaussian filters, and Discrete Cosine Transform. They used bi-clustering analysis to identify and associate code words with conceptual classes and then selected the most discriminative code words. Han et al. [35] compared the performance of sparse coding with classical kernel methods to classify normal brain tissue, cancer, and necrotic tissue. They found kernel methods performed better than sparse coding with respect to their database. Finally, several studies used neural networks to identify necrosis. For example, Han, Nayak et al. [36] decomposed histopathologic images into distinct areas by using a variation of Restricted Boltzmann Machine to classify patches from the National Institutes of Health’s Cancer Genome Atlas Archive, achieving accuracy >81% on two different databases. In a follow-up study, Chang et al. [33] used morphometric features and morphometric sparse code algorithms within the Spatial Pyramid Matching framework for classification of necrosis. They found that modeling the context of the morphometric information outperforms algorithms based on pixel or patch features. More recently, Chang et al. [34] proposed an unsupervised multi-stage feature learning algorithm to detect normal tissue, cancer, and necrosis. They used stacked Predictive Sparse Decomposition at different locations and scales, followed by a linear SVM classifier to “diagnose” two cancer types. Performance was highly promising at >91.89% accuracy on 2 publicly available cancer databases.

**Figure 1:**
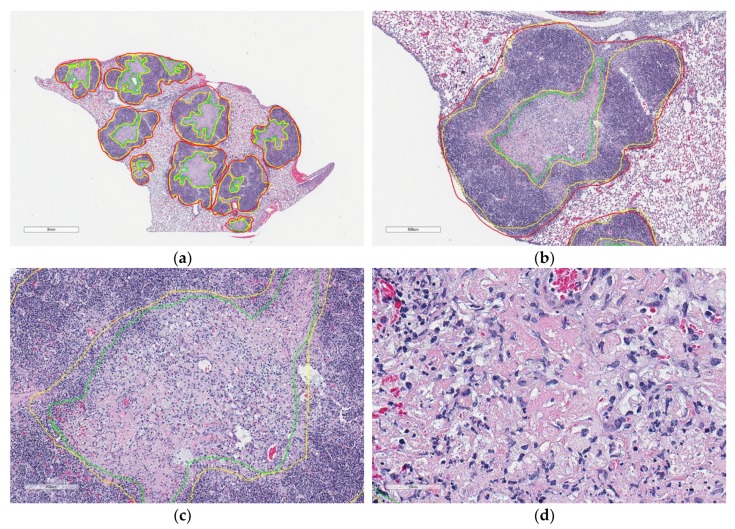
Microscopic appearances of lung granulomas with central cell-poor necrosis, and peripheral cellular and nuclear debris from a super-susceptible DO mouse infected with *M. tuberculosis*. Lung tissue was formalin-fixed, paraffin-embedded, sectioned at 5 μm, adhered to glass slides, stained with hematoxylin and eosin, and digitally scanned. The colored lines outline regions of interest: Granulomas (red) contain central cell-poor necrosis (green), and a layer of dense peripheral cellular and nuclear debris (yellow). (**a**) Lung magnified 10×. (**b**) Granuloma magnified 40×. (**c**) Central region of a granuloma magnified 100×. (**d**) Cell-poor necrosis magnified 400×.

The studies above focus on necrosis related to cancer; however, necrosis is also a host response to infection with pathogenic microorganisms including *M. tuberculosis*. Like humans diagnosed with pulmonary TB, granuloma necrosis in occurs in a fraction of Diversity Outbred (DO) mice and rarely in inbred laboratory strains of mice experimentally infected with *M. tuberculosis* [10,11,37]. Necrotic granulomas are found in >85% of super-susceptible DO mice and the histologic patterns include central cell-poor eosinophilic necrotic cores surrounded by pyknotic and karyorrhectic cellular debris (Figure 1), and a peripheral layer of neutrophils and macrophages admixed with few lymphocytes and plasma cells [11].

Our previous research segmented macrophage- and lymphocyte-rich regions of *M. tuberculosis* granulomas and normal lung tissue, while necrosis was not considered [12,17,38]. Because of the importance of necrosis in pulmonary TB diagnosis and pathogenesis, here our objective was to develop, train, validate and test 2D-TB, a supervised algorithm that detects necrosis in granulomas using color and texture features. To our knowledge, we are the first to publish automatic and accurate detection of cell-poor granuloma necrosis in an experimental model of pulmonary TB.

## 2. Materials and Methods

### 2.1. M. tuberculosis Infection of Diversity Outbred Mice, Lung Tissue Staining and Slide Scanning

Procedures were approved by Tufts Institutional Animal Care and Use Committee (IACUC), and by Tufts Institutional Biosafety Committee (IBC). The Cummings School of Veterinary Medicine (CSVM) is AAALAC-accredited; maintains a letter of Assurance with the Office of Laboratory Animal Welfare; and follows the Public Health Service Policy on Humane Care and Use of Laboratory Animals; the Guide for the Care and Use of Laboratory Animals; and the U.S. Government Principles for the Utilization and Care of Vertebrate Animals Used in Testing, Research, and Training. These studies were approved under IACUC protocol numbers G2012-53, G2015-33, and G2018-02; and IBC registrations GRIA04, GRIA10, and GRIA17. Female 8-week old DO mice from The Jackson Laboratory (Bar harbor, ME, USA) were housed in the Biosafety Levels 3 facility at the New England Regional Biosafety Laboratory (Tufts University, Cummings School of Veterinary Medicine, North Grafton, MA, USA). Mice were infected with 20 to 100 Colony Forming Units of *M. tuberculosis* Erdman using a CH Technologies nose-only exposure system, as previously described [11,12,13].

Following euthanasia, mouse lungs were inflated and fixed in 10% neutral buffered formalin, processed and embedded in paraffin, sectioned at 5 μm and stained with hematoxylin and eosin at Tufts University, Cummings School of Veterinary Medicine, Core Histology Laboratory (North Grafton, MA, USA). Hematoxylin and eosin are standard histochemical stains used for diagnostic and research pathology. It is the most familiar stain for pathologists. The hematoxylin stains nuclei and structures rich in nucleic acids gray to blue to dark purple. The eosin stains protein-rich regions of cells and tissues various shades of pink. A limitation to hematoxylin and eosin staining in our context is that *M. tuberculosis* bacilli are not stained by either hematoxylin or eosin. Therefore, bacilli are not visible within the cells or within the regions of necrosis.

Glass slides containing hematoxylin and eosin stained lung sections were magnified 400 times and digitally scanned using Aperio ScanScope (Leica Biosystems Inc., Buffalo Grove, IL, USA) at The Ohio State University’s Comparative Pathology and Mouse Phenotyping Shared Resources Core Facility (Columbus, OH, USA).

### 2.2. Image Database, Annotations, Validation

Fourteen whole slide images ranging in size from 100,000 × 50,000 pixels to 160,000 × 90,000 pixels were obtained from the Department of Infectious Disease and Global Health at the Tufts University. A board-certified veterinary pathologist (GB) manually annotated 29 granulomas, 40 regions of cellular and nuclear debris, and 136 regions of cell-poor necrosis in lungs from 8 different super-susceptible DO mice. From the annotations on 4 slides (8 different super-susceptible DO mice) 23.5% were used for algorithm training and 76.5% were used for 10-fold cross validation. Annotations on the remaining 10 slides, containing 1–2 lung lobes from 10 different super-susceptible DO mice (which were not used in training) were used to test accuracy as compared to a board-certified veterinary pathologist (GB).

### 2.3. Measures of Accuracy to Assess 2D-TB Performance

Agreement of cell-poor necrosis between 2D-TB and the pathologist were determined by many methods: Sensitivity, Specificity, Positive Prediction Value, Dice similarity measurement and Spearman correlation for area (μm). For Dice similarity [13], necrotic areas detected by 2D-TB were marked on whole slide images. An expert pathologist (GB) then classified the marked areas into four categories: ‘Agree’, ‘Mostly Agree’, ‘Disagree’ and ‘Uncertain’, ‘Agree’ or ‘Mostly Agree’ were recorded as True Positive (TP). ‘Disagree’ labels were recorded as False Positive (FP). ‘Uncertain’ labels were recorded as TP if a matched tissue pair (i.e., a serial section from the same lung) on the whole slide image was classified as ‘Agree’ at the same location; otherwise, discordant regions were recorded as FP.

Dice similarity measure (Equation (1)), sensitivity (SN) and Positive Predictive Value (PPV) measures (Equations (2) and (3)) are calculated as follows:

(1) $$D\left(GT,Computed\right)=\frac{\left|GT\cap^{\;}Computed\right|}{\frac{1}{2}\left(\left|GT\right|+\left|Computed\right|\right)}=\frac{2\times TP}{\left(FN+TP\right)+\left(TP+FP\right)}$$

(2) $$SN=\frac{TP}{TP+FN}$$

(3) $$PPV=\frac{TP}{TP+FP}$$

where GT indicates Ground Truth and *Computed* indicates detected results. To determine quantify error, we calculated the misclassification rate (MCR) as follows (Equation (4)):

(4) $$MCR=\frac{\mathrm{number}\;\mathrm{of}\;\mathrm{misclassified}\;\mathrm{images}}{\mathrm{total}\;\mathrm{number}\;\mathrm{of}\;\mathrm{images}}$$

GraphPad Prism 8 (San Diego, CA, USA) was used to compare the area (μm^2^) of cell-poor necrotic regions determined by 2D-TD and the same regions manually annotated by a pathologist. Spearman correlation coefficients were calculated and identified as very weak (0–0.19), weak (0.20–0.39), moderate (0.40–0.59), strong (0.60–0.79), or very strong (0.80–1.0) and considered statistically significant if *p* < 0.05.

### 2.4. Algorithm Overview and Implementation

**Figure 2:**
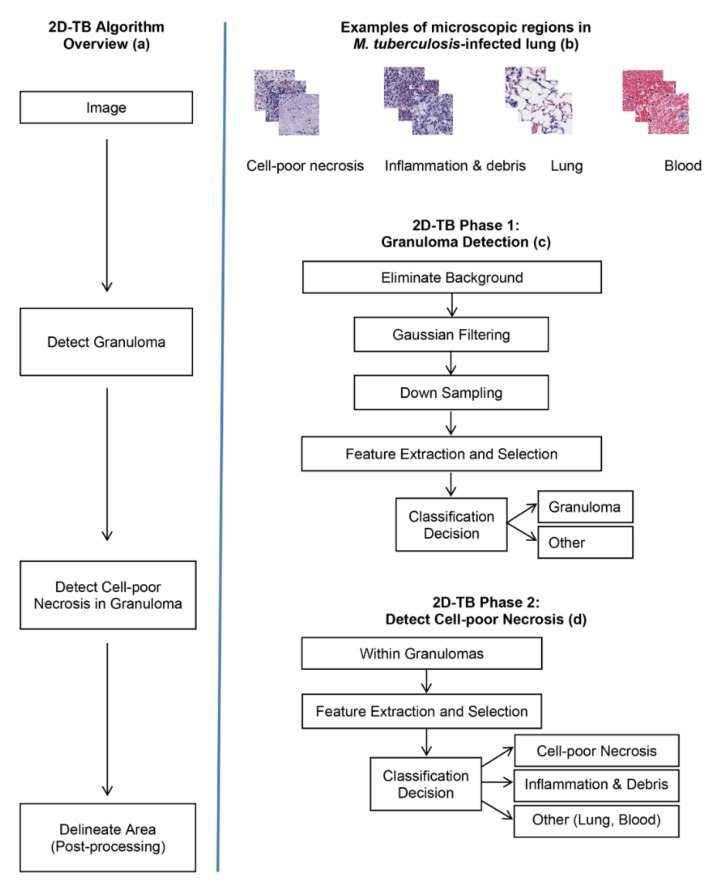
Schematic overview of 2D-TB. (**a**) Overview in block diagram. (**b**) Microscopic appearance of visually distinct regions in *M. tuberculosis*-infected lung: Cell-poor necrosis; inflammation and debris; normal lung tissue; and blood, magnified 400×. (**c**) 2D-TB phase 1: Granuloma detection in block diagram. (**d**) 2D-TB phase 2: Detection and classification of cell-poor necrosis within granulomas.

Figure 2a shows an overview of 2D-TB that uses visually distinct regions (Figure 2b) from *M. tuberculosis-*infected lung. In phase 1 of 2D-TB (Figure 2c), a learning model detects granulomas on low resolution images (64:1). This reduces the computational complexity. In phase 2 of 2D-TB (Figure 2d), high resolution images within the detected granuloma regions are searched for cell-poor necrosis. This increases specificity. As a post-processing step, nearby necrotic areas are merged using a connected component analysis to mark regions and determine the area. Adjacent pixels detected as necrosis are labelled and areas more than a defined threshold (per the approximations of the pathologist taken as 1000 pixel) are delineated as necrotic areas.

**Figure 3:**
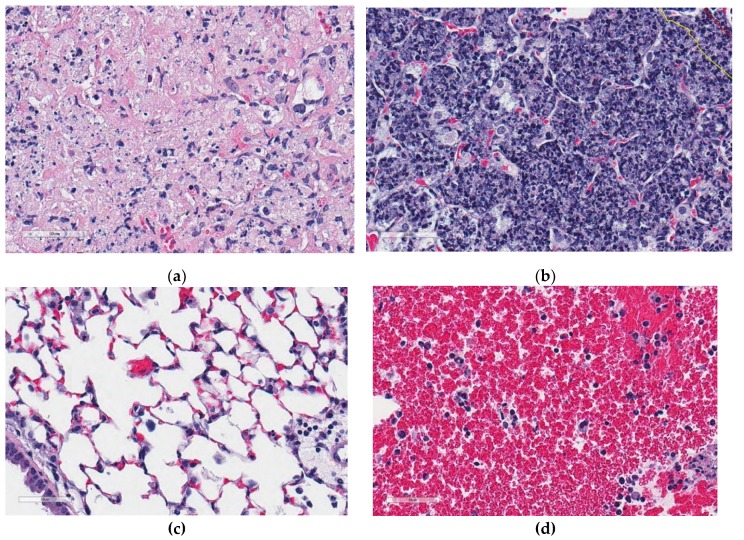
Examples of visually distinct regions from hematoxylin and eosin stained lung sections from super-susceptible *M. tuberculosis-*infected DO mice. (**a**) Cell-poor necrosis; (**b**) Inflammation and cellular debris; (**c**) Lung tissue; (**d**) Blood. Regions are magnified 400×.

The algorithms were implemented in Matlab^®^ (Natick, MA, USA). Training was performed using visually distinct regions from hematoxylin and eosin stained lung tissue from *M. tuberculosis-*infected super-susceptible Diversity Outbred mice. Figure 3 shows examples of training region types: Cell-poor necrosis (Figure 3a); inflammation and cellular debris (Figure 3b); lung tissue (Figure 3c) and blood (Figure 3d).

### 2.5. Automatic Granuloma Detection

Prior to granuloma detection, background is eliminated using an experimentally determined threshold value (235). The variation in the threshold value affects the estimation of the tissue section borders but does not impact the downstream process and the performance parameters.

**Figure 4:**
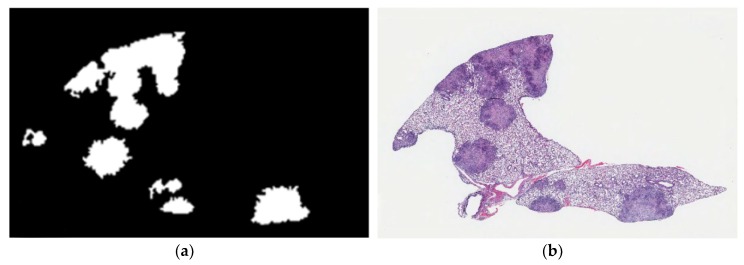
2D-TB phase 1 output detects granulomas from other non-granuloma regions in *M. tuberculosis-*infected lung from DO mice. (**a**) An example of the granuloma detection (white) is compared to (**b**) appearance of the same lung granulomas in its hematoxylin and eosin stained counterpart. Magnification is 10×.

Linear kernel SVM, Gaussian kernel SVM, decision tree and random forest classifiers were used to differentiate granulomas from normal lung tissue. To detect granulomas and distinguish from other, non-granuloma regions, we extracted 740 regions sized 512 × 512 pixels from lung tissue sections. These regions belonged to two classes: granuloma and other (non-granuloma). To reduce the computational cost, we used low-resolution (16:1) images in size 32 × 32 pixels to segment granulomas both in training and test images. In the training phase of granuloma region detection, R, G and B color channel values of training data were smoothed by a Gaussian filter (σ = 4) and followed by 16:1 down sampling. From the down-sampled image, features were extracted as described in Section 2.6. Figure 4a shows the granuloma detection output from phase 1 compared to the same hematoxylin and eosin stained image in Figure 4b. In phase 1, we noted that normal tissue was occasionally determined as granuloma. This occasional error in specificity, however, was deemed acceptable because any potential necrotic regions missed in phase 1 could not be recovered in phase 2.

### 2.6. Color Features and Derived Images

Granulomas and cell-poor necrosis exhibit unique color and texture features compared to other regions in the lung. Expert pathologists recognize granulomas (Figure 1b) by their blue-purple color due to hematoxylin staining with variable, central lighter pink cell-poor necrosis (Figure 1c,d) due to eosin uptake. Adjacent lung tissue (Figure 3c) appears as a white mesh. Blood (Figure 3d) is recognized as large regions of red due to dense accumulations of red blood cells. We modeled these color and texture features in terms of relative variations in R, G, B color channels at different pixel distances. These features are designed to be mimic joint probabilities of relative color channel values, ratios of color channel difference values, and contribution of each color channel values onto the intensity image. Since the intensity of an image resembles perceived luminance of human vision, each perceived color has a different hue value. Therefore, we used gamma-corrected combination of color channel values.

The intensity value of a pixel is defined by performing weighted sum of the R, G, and B color channel components (Equation (5)) that is commonly used for grayscale conversion.

*I*_gray_ = 0.299 × *Red* + 0.587 × *Green* + 0.114 × *Blue* (5)

Each value of the weighted color channels is defined separately (Equations (6)–(8)).

*R*’ = 0.299 × *Red* (6)

*G*’ = 0.587 × *Green* (7)

*B*’ = 0.114 × *Blue* (8)

To measure the contribution of each color channel onto the intensity image, we obtained derived images as the ratios of color channel values. Derived images capture the relative color differences and were needed because constant thresholds for color differences could not accommodate staining intensity differences. The absolute differences were included as the absolute value of variation rather than the sign. For all derived images, mean, standard deviation and entropy values were calculated.

### 2.7. Detection of Cell-Poor Necrosis in Granulomas Using Machine Learning

**Figure 5:**
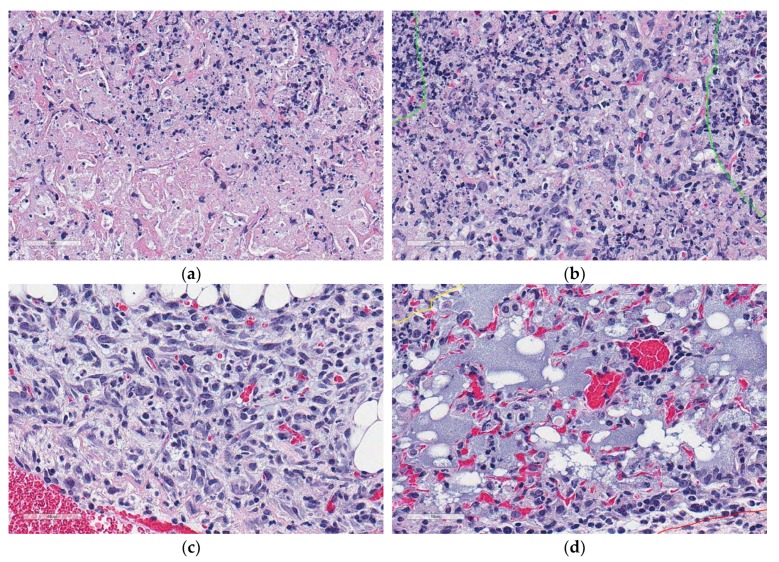
Examples of regions of cell-poor necrosis and other non-necrotic regions in *M. tuberculosis* lung granulomas. (**a**,**b**) show examples of cell-poor necrosis compared to (**c**,**d**) which are non-necrotic regions, and contain viable cells, blood, and other non-cellular material (fluid). Magnification is 400× from hematoxylin and eosin stained lung.

Phase 2 of 2D-TB detects regions of cell-poor necrosis within granulomas. Cell-poor necrosis (Figure 5a,b) can be differentiated from non-necrotic regions (Figure 5c,d) in granulomas by color and texture which reflects the loss differential staining and relatively low density of viable calls with intact nuclei, and reduced amount of blood. Necrotic areas are various hues of pink primarily due to cellular, tissue, and blood proteins, with sparse intact nuclei and a relatively homogeneous texture.

**Figure 6:**
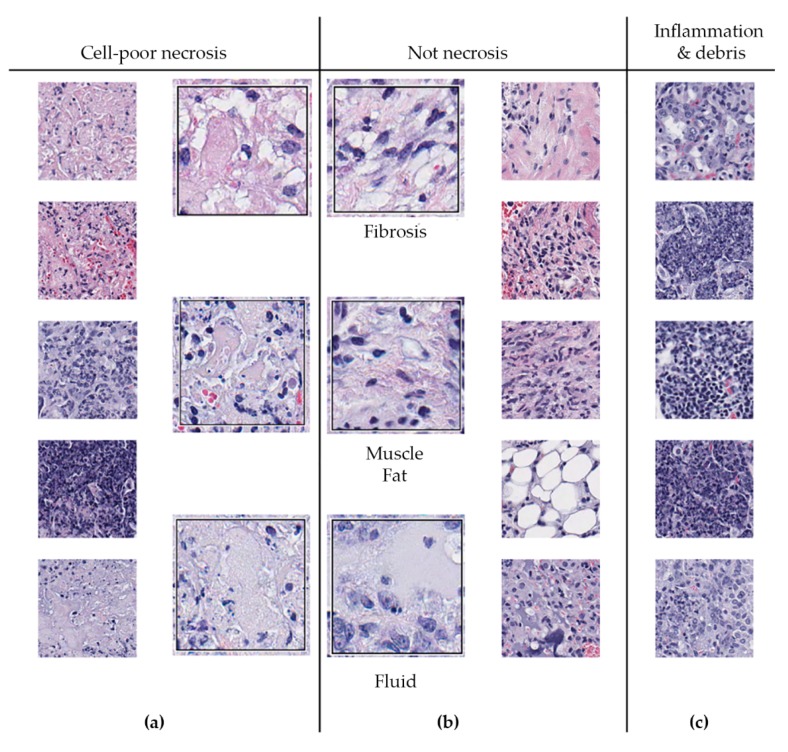
Regions within *M. tuberculosis-*infected lungs that may resemble cell-poor necrosis. (**a**) Shows examples of cell-poor necrosis; (**b**) Fibrosis, smooth muscle, fat, and fluid can resemble necrosis as can (**c**) regions of inflammatory cells and debris within granulomas. Small images are magnified 100×; Larger images are magnified 400×. A board-certified veterinary pathologist (GB) confirmed the interpretations.

We also observed that regions of cell-poor necrosis (Figure 6a) may resemble fibrosis, smooth muscle, fat, and fluid-filled alveoli (Figure 6b). Granulomas may also contain regions with more viable inflammatory and immune cells, and regions of abundant cellular and nuclear debris (Figure 6c) that are also within granuloma can also resemble necrosis. These regions need to be distinguished from necrosis.

**Figure 7:**
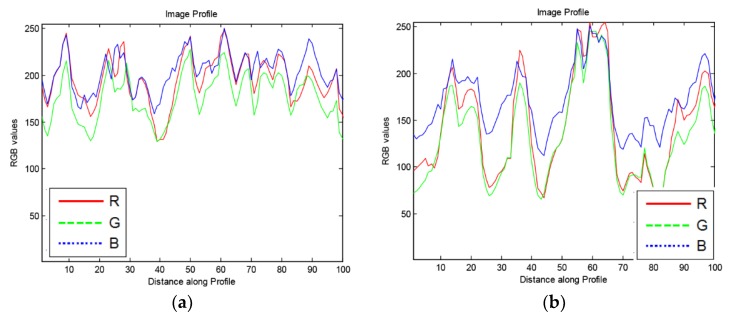

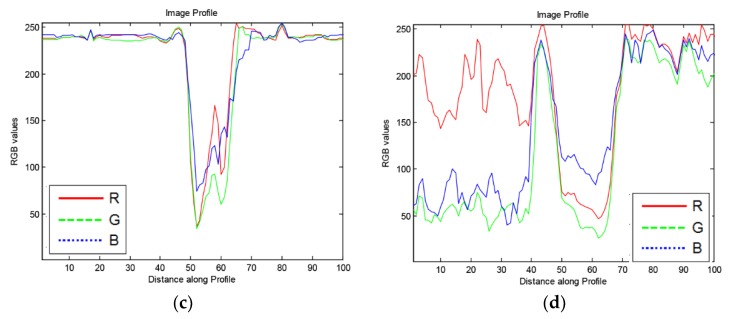
Examples of 100× 1-pixel R, G, B image profiles of different regions from *M. tuberculosis* infected, hematoxylin and eosin-stained, lung tissue from a super-susceptible DO mouse: (**a**) Cell-poor necrosis; (**b**) Inflammatory cells and nuclear debris; (**c**) Lung tissue; and (**d**) red blood cells.

To avoid detecting regions that may be confused with cell-poor necrosis (Figure 6), we obtained 100-pixel horizontal image profiles from different regions and examined the color profiles. Figure 7 shows representative profiles. In regions of cell-poor necrosis (Figure 7a), R, G, and B color channel values are close to each other with generally higher and less B color channel than the values of inflammatory cells and nuclear debris (Figure 7b). In Figure 7c, the lung tissue R, G, B color channel values are close to each other and have relatively high values (e.g., 230). As expected, in areas with blood cells (Figure 7d), the R color channel values are higher than G and B color channel values.

In phase 2 of 2D-TB, regions of cell-poor necrosis are detected by searching high-resolution (40× or 1:1) whole slide images within predefined granuloma regions mapped onto the high-resolution image. In this phase, derived images marked with ‘*’ in Table 1 were obtained by using 512 × 512 pixel-sized patches from the predefined regions.

Statistical parameters in Table 2 were then calculated and used as features to be classified by a linear kernel SVM classifier, which labeled each patch as ‘necrosis’ or ‘not necrosis’. To train and test a classifier, we extracted 690 samples of a size of 512 × 512 pixels at a 1:1 resolution. Each sample image was divided into four sub images (256 × 256 pixels), resulting in 2760 image samples that were used for training and testing purposes. To capture a higher level of detail, we used high-resolution images to segment necrosis.

### 2.8. Features for Detection of Cell-Poor Necrosis

**Figure 8:**
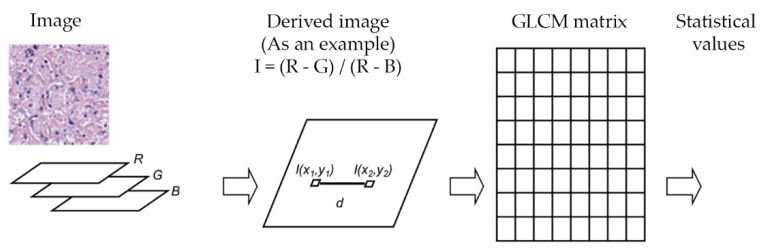
Texture information extraction to aid detection of cell-poor necrosis within lung granulomas due to *M. tuberculosis*. A gray-scale image was produced by the derived image calculations. Then, a GLCM matrix was computed with select summary statistics features (see Table 1).

Linear kernel SVM, Gaussian kernel SVM and decision trees were evaluated to differentiate cell-poor necrosis from inflammatory cells and nuclear debris and from other types of cells/regions in lung tissues. We trained a learning model to identify three different classes: Cell-poor necrosis; Inflammatory cells and nuclear debris; and ‘Other’ (i.e., normal lung, fibrosis, fluid-filled alveoli, fat, smooth muscle, and blood) using color features as previously described, and texture features. To exploit texture information, statistical values of horizontal gray level co-occurrence matrices (GLCM) [11,12] of derived images were used. The texture information was obtained from both GLCM and statistical values of derived images *I*. Relative frequency matrix of two separate neighbors at certain pixel distance *d* was calculated. GLCM matrices are constituted in only one angle (zero degree) with a function of distance *d* to reduce computation times. Figure 8 and Table 1 show the feature definitions and 10 statistical features extracted from derived images and the average and standard deviation features calculated from derived images.

## 3. Results

By using color and texture features to automatically and accurately detect, segment and quantify cell-poor granuloma necrosis, we developed, trained, and performance-tested 2D-TB. For phase 1 (granuloma detection), mean, standard deviation and entropy values of nine different derived images were calculated. To differentiate granulomas from normal lung tissue; Linear kernel SVM, Gaussian kernel SVM, decision tree and random forest classifiers were used. For phase 2 (detection of cell-poor necrosis) to differentiate cell-poor necrosis from other types of cells/regions in lung tissues; linear kernel SVM, Gaussian kernel SVM and ensemble of decision trees were evaluated.

Table 3 summarizes the selected features and MCR results for 4 different classifiers to distinguish cell-poor necrosis from other. To measure performance, 10-fold cross-validation was applied on 10 whole slides magnified 400×. The best performance (lowest misclassification rate) was obtained with SVM classifiers using 2 features. We favor the linear kernel SVM because of its lower computational complexity compared to Gaussian kernel that nonlinearly maps samples into a higher dimensional space. In our data, SVM classifiers had the lowest MCR rates.

For phase 2, we also investigated the effect of gray-level co-occurrence matrix (GLCM) distance parameter on algorithm performance (Table 4). The lowest MCR was obtained at 5-pixel distance for linear SVM classifier method. Therefore, we selected a 5-pixel distance to distinguish regions of cell poor necrosis from other regions.

**Figure 9:**
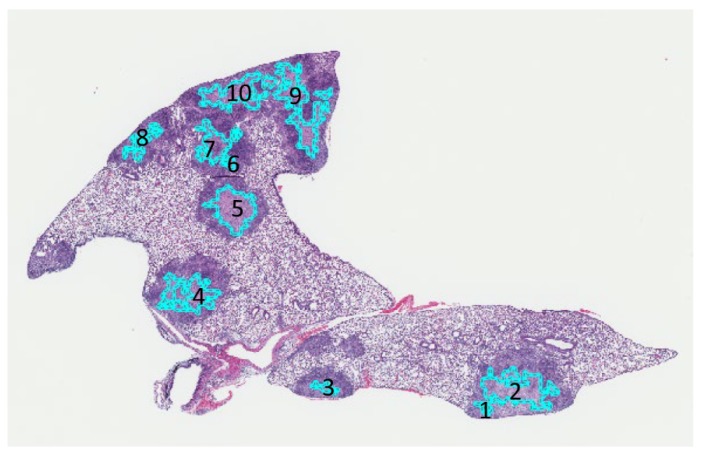
2D-TB automatically detected 10 cell-poor necrotic regions outlined in teal within *M. tuberculosis* granulomas, mapped back onto a hematoxylin and eosin stained lung section.

Figure 9 shows output by 2D-TB to automatically detect and mark 10 different cell-poor necrotic regions within *M. tuberculosis* granulomas on one section of hematoxylin and eosin stained lung. Table 5 shows the written feedback from a board-certified pathologist (GB) for each region.

Table 6 tabulates the TP, FP, PPV and Dice similarity values after the expert pathologist’s evaluation of annotations by 2D-TB for 292 necrotic regions in 10 different super-susceptible DO mice during independent testing. For the computer annotated slides, sensitivity values were considered 100% because no areas were marked by the pathologist as missed by 2D-TB.

**Figure 10:**
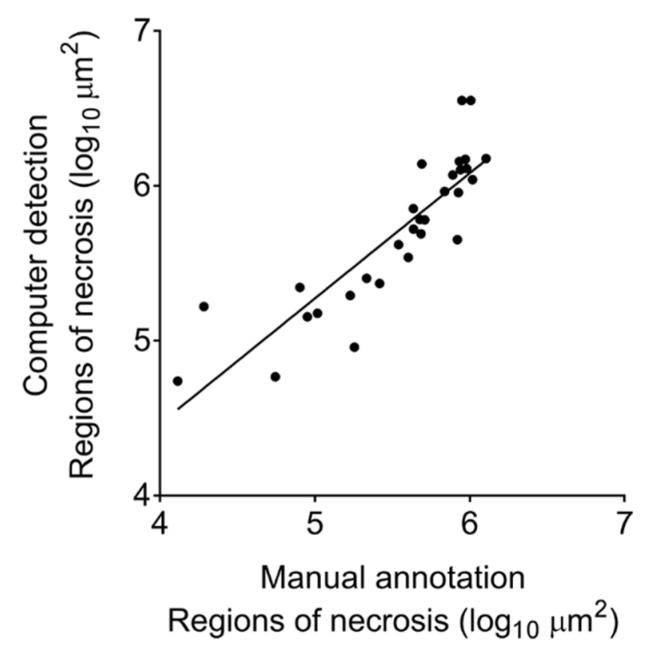
Correlation between the size of cell-poor necrosis regions within *M. tuberculosis* granulomas as measured by the two-phase algorithm, 2D-TB, and the same regions manually annotated and measured by a board-certified pathologist (GB).

Figure 10 demonstrates the very strong, positive, statistically significant correlation (Spearman *r* = 0.93, *p* < 0.0001) between the area (μm^2^) of 30 different regions of cell-poor necrosis within granulomas that were automatically detected by 2D-TB and the same regions manually annotated by a board-certified veterinary pathologist (GB).

## 4. Discussion

Objective detection of lesions within histopathological images, such as cell-poor necrosis within *M. tuberculosis* granulomas in pulmonary TB is important in diagnostic and research pathology. By automating detection of granuloma necrosis, we produced a tool for efficient and unbiased detection and quantification cell-poor granuloma necrosis. In future studies, 2D-TB will be used for rigorous comparisons across study groups that may improve our understanding of the mechanisms that result in differential susceptibility to *M. tuberculosis in vivo.* We anticipate this will provide insight into processes that have primarily been studied in vitro [10,30] with few exceptions [39].

Detecting cell-poor necrosis automatically is challenging because of the high morphologic variability of tissues within each class (e.g., Figure 3, Figure 5, Figure 6). Here, we developed an accurate 2-phase algorithm, designated 2D-TB because it functions on 2-dimensional images in 2 phases. 2D-TB detects and delineates cell-poor necrosis in granulomas based on the color and texture features with high accuracy. We used multiple methods to determine agreement with an expert veterinary pathologist (GB), including Dice similarity measure, sensitivity, specificity, and positive predictive value. Agreement was >91% and correlation was very high, positive, and statistically significant. Considering the variability within the necrosis class, in our future studies, we will divide necrosis class into two or more classes to better capture the variance.

2D-TB uses relative value statistics of R, G, and B color channel pixel intensity combinations as color features to detect and discriminate different regions within the lungs and within granulomas of super-susceptible, *M. tuberculosis-*infected DO mice. We used low-resolution images in the preprocessing phases such as finding the region of interest to improve efficiency. More specifically, we used low-resolution images to eliminate the background area, to detect the granulomas, and then to map back onto the high-resolution image for further determination of cell-poor necrosis. After a background mask was applied, images were subdivided into small square “tiles” to process the lung tissue regions on the whole slide images. This divided large images into small sub-images, decreasing the computation intensity. Next, the tiles were classified as granuloma or other, non-granuloma. The granuloma regions were then fed into the classifier to determine whether images were cell-poor necrosis, inflammation and nuclear debris, or other. Finally, the detected regions of cell-poor necrosis were stitched together by component analysis.

We focused on extracting the R, G, and B color channel information and texture information. We hypothesized that hematoxylin and eosin stain color combinations from different tissues could be distinguished by using relative R, G, and B parameters, following the pathologist’s observation that granulomas and necrosis contain primarily pink and to a lesser extent purple to bluish tones. We believe that this method may also be useful for other tissues with different stain absorptions, for example skin. To improve the effectiveness of 2D-TB, we added different texture features that reflect different aspects of the visual content. For a follow-up to this study, necrotic areas could be further classified based on texture features, for example, detection of individual cell necrosis and pyknotic debris.

Although the misclassification rates were generally quite low, and no necrotic regions were missed, 2D-TB occasionally misclassified regions of bronchiolar airway epithelium with or without inflammation as granulomas or as cell-poor necrosis. These were false positives, easily corrected by a pathologist review. There were rare regions of uncertainty that 2D-TB and pathologist both identified as necrosis at a low magnification but review at high magnification yielded regions of mixed neutrophil and macrophage inflammation. These regions may be early manifestations of necrosis that do not fit a distinct morphology.

## 5. Conclusions

Overall, our new cell-poor necrosis detecting algorithm, 2D-TB, detects cell-poor granuloma necrosis in an experimental model of pulmonary TB with high accuracy and strong agreement with an expert veterinary pathologist. More specifically, we obtained an average sensitivity of 100.0% and Dice value of 95.5% for 10 slides containing 13 pairs of lung lobes. Future studies will include performance testing on other animal models of granuloma necrosis and samples from human patients with pulmonary TB to build a larger database and further validate the results.

## Figures and Tables

**Table 1:** Derived images and statistical parameters. The ‘*’ marks images obtained from low-resolution of 512 × 512 pixel-sized patches from these tissue locations.

Feature	Derived Images (*I*)	Statistical Parameters [11,12]
Color channel difference ratios	(R-B)/(B-G) (R-G)/(B-G) (R-G)/(R-B) *	Contrast Correlation Cluster Shade Energy Entropy Homogeneity Sum average Sum variance Sum entropy Information measure of correlation
Weighted color channel ratios	(R’ + B’)/(B’ + G’) (R’ + G’)/(B’ + G’) (R’ + G’)/(R’ + B’) * (R’ + B’)/(R’ + B’ + G’) (R’ + G’)/(R’ + B’ + G’) (B’ + G’)/(R’ + B’ + G’)	
Color channel ratios	R/G G/B * R/B *	
Gray level image	I = R’ + G’ + B’ *	
Derived Images (*I*)		Statistical parameters
(R-B)/(B-G) * (R-G)/(B-G) (R-G)/(R-B) * (R’ + B’)/(B’ + G’) * (R’ + G’)/(B’ + G’) (R’ + G’)/(R’ + B’) *	(R’ + B’)/(R’ + B’ + G’) * (R’ + G’)/(R’ + B’ + G’) (B’ + G’)/(R’ + B’ + G’) * R/G * G/B R/B *	Mean Standard Deviation

**Table 2:** Derived images and statistical parameters. To ensure non-zero division, a small value, 0.01 value was added. The ‘*’ marks images obtained from low-resolution of 512 × 512 pixel-sized patches from these tissue locations.

Derived Images	Statistical Parameters
R’/(R’ + B’ + G’)	Mean Standard Deviation Entropy
R’/(R’ + B’ + G’)	
G’/(R’ + B’ + G’)	
B’/(R’ + B’ + G’)	
R/G	
G/B	
R/B	
abs((R-B)/(B-G)) *	
abs((R-G)/(B-G)) *	
abs((R-G)/(R-B))	

**Table 3:** Classifier results for 2D-TB.

Classifier	SVM Linear Kernel	SVM Gaussian Kernel	Decision Tree	Random Forest
Selected features	Mean absolute of (R-G)/(B-G) Mean absolute of (R-B)/(B-G)	Mean absolute of (R-G)/(B-G) Standard deviation of R’/(R’ + G’ + B’)	Mean absolute of (R-G)/(B-G)	Mean absolute of (R-G)/(B-G) Mean of G’/(R’ + G’ + B’)
Misclassification rate (%)	0.27	0.27	0.40	0.40

**Table 4:** Classifier and misclassification rates for phase 2 with GLCM distance.

Distances (Pixels)	SVM Linear Kernel	SVM Gauss Kernel	Ensemble
5	% 7.06 (17 Features)	% 10.03 (22 Features)	% 8.11 (14 Features)
10	% 8.33 (13 Features)	% 13.44 (20 Features)	% 9.09 (12 Features)
15	% 7.75 (16 Features)	% 15.76 (10 Features)	% 9.92 (11 Features)
20	% 8.55 (14 Features)	% 14.85 (11 Features)	% 9.74 (11 Features)

**Table 5:** An example of the board-certified pathologist’s (GB) feedback on 10 regions of cell-poor necrosis detected by 2D-TB, shown above in Figure 9.

Region Detected by 2D-TB	Pathologist Comments
1	Mostly agree
2	Agree
3	Agree
4	Agree
5	Agree
6	Agree
7	Agree
8	Agree
9	Agree
10	Agree

**Table 6:** Performance results of 2D-TB to detect cell-poor necrosis within *M. tuberculosis* granulomas compared to a board-certified veterinary pathologist (GB).

Section No.	Mouse/Lung No.	No. Necrotic Regions	TP	FP	PPV (%)	Dice Similarity (%)
1	10/1	9	6	3	66.7	80.0
2	10/2	18	17	1	94.4	97.1
3	13/1	41	41	0	100.0	100.0
4	13/2	45	44	1	97.8	98.9
5	109	13	13	0	100.0	100.0
6	118	13	13	0	100.0	100.0
7	144/1	8	8	0	100.0	100.0
8	144/2	14	12	2	85.7	92.3
9	149	30	29	1	96.7	98.3
10	156	8	8	0	100.0	100.0
11	158	41	30	11	73.2	84.5
12	173	26	24	2	92.3	96.0
13	164	26	23	3	88.5	93.9
Average:			20.62	1.85	91.94	95.46
Standard Deviation		12.85	12.00	2.85	10.48	6.19
